# Autism spectrum disorder diagnosis in adults: phenotype and genotype findings from a clinically derived cohort

**DOI:** 10.1192/bjp.2019.30

**Published:** 2019-11

**Authors:** Jack F. G. Underwood, Kimberley M. Kendall, Jennifer Berrett, Catrin Lewis, Richard Anney, Marianne B. M. van den Bree, Jeremy Hall

**Affiliations:** 1 Clinical Research Fellow, Neuroscience and Mental Health Research Institute, Cardiff University, UK; 2 Wellcome Trust Clinical Research Fellow, MRC Centre for Neuropsychiatric Genetics and Genomics, Cardiff University, UK; 3 Trainee Clinical Psychologist, Neuroscience and Mental Health Research Institute, Cardiff University, UK; 4 Research Associate, National Centre for Mental Health, Cardiff University, UK; 5 Senior Lecturer (Bioinformatics), MRC Centre for Neuropsychiatric Genetics and Genomics, Cardiff University, UK; 6 Professor of Psychological Medicine, MRC Centre for Neuropsychiatric Genetics and Genomics, Cardiff University, UK; 7 Director and Research Theme Lead, Neuroscience and Mental Health Research Institute, Cardiff University, UK

**Keywords:** Autistic spectrum disorders, genetics, social functioning

## Abstract

**Background:**

The past decade has seen the development of services for adults presenting with symptoms of autism spectrum disorder (ASD) in the UK. Compared with children, little is known about the phenotypic and genetic characteristics of these patients.

**Aims:**

This e-cohort study aimed to examine the phenotypic and genetic characteristics of a clinically presenting sample of adults diagnosed with ASD by specialist services.

**Method:**

Individuals diagnosed with ASD as adults were recruited by the National Centre for Mental Health and completed self-report questionnaires, interviews and provided DNA; 105 eligible individuals were matched to 76 healthy controls. We investigated demographics, social history and comorbid psychiatric and physical disorders. Samples were genotyped, copy number variants (CNVs) were called and polygenic risk scores were calculated.

**Results:**

Of individuals with ASD, 89.5% had at least one comorbid psychiatric diagnosis, with depression (62.9%) and anxiety (55.2%) being the most common. The ASD group experienced more neurological comorbidities than controls, particularly migraine headache. They were less likely to have married or be in work, and had more alcohol-related problems. There was a significantly higher load of autism common genetic variants in the adult ASD group compared with controls, but there was no difference in the rate of rare CNVs.

**Conclusions:**

This study provides important information about psychiatric comorbidity in adult ASD, which may inform clinical practice and patient counselling. It also suggests that the polygenic load of common ASD-associated variants may be important in conferring risk within the non-intellectually disabled population of adults with ASD.

**Declaration of interest:**

None.

Autism spectrum disorder (ASD) is a group of neurodevelopmental disorders characterised by persistent difficulties in social interaction and communication, as well as restricted interests, stereotypic behaviours and resistance to change.^1^ Epidemiological studies report a prevalence of ASD in the general population of around 1%, with a male:female ratio of approximately 3:1.^2^^–^^5^ The majority of studies of ASD have been carried out in paediatric populations and have included individuals with intellectual disability. The limited studies in individuals diagnosed with ASD as adults have shown that adults with ASD experience significant disadvantage in employment, social relationships and quality of life.^6^^,^^7^ ASD has also been associated with increased lifetime psychiatric comorbidity.^3^^,^^8^^–^^10^ Research has particularly focussed on anxiety and depression, where a meta-analysis by Hollocks *et al* demonstrated lifetime rate estimates of 42% and 37%, respectively.^11^ This analysis found considerable heterogeneity between the studies, and no studies examining comorbidity in non-clinical samples of adults with ASD.^11^ There has been very little research reporting lifetime outcomes for individuals diagnosed with ASD as adults. This information has the potential to inform specialist service development and tailor clinical care. Advancing knowledge in this area is important because since the Autism Act 2009 and recommendations in the National Institute for Health and Care Excellence Guidelines of 2012, many adult ASD diagnostic services have been set up across the UK, although provision remains sporadic.^12^^–^^14^ Furthermore, although some adult services now offer genetic testing for individuals diagnosed with ASD, little is known about the genetic characteristics of adults presenting with ASD. Studies of predominantly paediatric ASD populations have shown a substantial contribution of both rare copy number variants (CNVs) and polygenic burden of common variants to risk for ASD. Rare CNVs are reported to occur in 10–15% of childhood ASD cases, which has encouraged formal genetic testing in childhood ASD.^15^^,^^16^ However, it is not known whether similar rates are seen in individuals presenting to adult diagnostic services. In this study we examine the demographic, social, psychiatric and physical health characteristics of a cohort of individuals presenting with ASD in adulthood compared with a healthy control population from the same source databank. We also report the rates of neurodevelopmental CNVs and polygenic burden of common variants associated with ASD in this sample.

## Method

### Sample

Data was obtained from the National Centre for Mental Health (NCMH, https://www.ncmh.info), a Welsh Government-funded Research Centre that investigates neurodevelopmental, adult and neurodegenerative psychiatric disorders across the lifespan. Participants were recruited using a variety of systematic approaches in primary, secondary and tertiary healthcare services, including the identification of potential participants by clinical care teams, screening of clinical notes and the use of disease registers. The majority of participants in our sample were recruited via specialist diagnostic services. Non-systematic recruitment approaches included advertising in local media, placing posters and leaflets in National Health Service waiting areas, liaising with voluntary organisations and contacting individuals enrolled in previous studies within the Institute of Psychological Medicine and Clinical Neurosciences, Cardiff, UK. To allow comparisons, the cohort includes control participants who self-report no experiences of any mental health disorder. All adult participants included in this study provided written informed consent for recruitment into the NCMH. Trained research assistants administered a brief standardised interview assessment to consenting participants to ascertain details related to the participant's personal and family history of mental health experiences, including any comorbidity, past and current medication use, sociodemographic information including employment and education, physical health diagnoses and any substance misuse. A sample of venous blood or saliva was taken for genetic and other analyses. Participants were given a pack of standardised self-report questionnaires to complete and return to the research team by post after the initial assessment. Confirmation and further information regarding the primary diagnosis of ASD was obtained from clinical records where appropriate consent had been obtained to do so.

To date, 10 870 individuals have been recruited to the NCMH. At the point of initial search in June 2016, the database included approximately 6600 participants, of whom 172 held a primary self-report diagnosis of Asperger syndrome, ASD or autism and no self-report comorbid intellectual disability, and were potentially eligible for inclusion. On case-note review, 67 out of 172 participants were excluded, predominantly because of loss of contact with the NCMH to confirm diagnosis (*n* = 37; full details in Supplementary Material, available at https://doi.org/10.1192/bjp.2019.30). The remaining 105 individuals were all confirmed to have an ASD diagnosis consistent with ICD-10 criteria by case-note review, with no evidence of recorded intellectual disability and with a first diagnosis of ASD made by secondary care clinicians assessing in a diagnostic role when the participant was over 18 years of age.^1^ Seventy-six controls were randomly selected from a derived cohort matched pairwise on age (within 5 years and over 18 years), ethnicity and gender from the NCMH database. Control participants were selected from individuals in the NCMH database with no current or previous self-reported difficulties with mental health and no psychotropic medication usage. A favourable ethical opinion was received from the Wales Research Ethics Committee 2 on 25 November 2016 for the NCMH, and through internal NCMH applications for this study.

### Measures

#### Demographic information

Demographic data was collected by interview and questionnaire. Biological offspring was recorded as a binary yes/no variable and with free-text number and biological age of children, recorded in 101 of the 105 participants with ASD and all controls. Lifetime marriage and cohabitation was recorded as a binary yes/no variable, recorded in 97 of the participants and all 76 controls. Current profession responses were multiple-choice skill level (see Supplementary Material for categorisation), reduced to ‘currently in work’/‘currently not in work’ for our analysis and recorded for 97 participants with ASD and 73 controls.

#### Comorbidities

Physical health comorbidities were established with a multiple-choice list of 22 common physical diagnoses selected for collection at the inception of the NCMH (Supplementary Material), reduced to clinical system categories for comparison. Lifetime mental health comorbidities were established with a multiple-choice list of 37 common psychiatric diagnoses, as reported in the Supplementary Material. By definition the control group had no psychiatric comorbidity for comparative analysis, thus preventing comparative analysis of mental health comorbid rates. Fifty-nine participants with ASD and 53 control participants completed the Beck Depression Inventory (BDI),^17^ providing information on current mood. Information on lifetime psychotropic medication usage was collected by medication class and provided by between 93 and 95 participants with ASD, dependent upon the question response rate.

#### Substance use

Individuals reported on problems encountered in their lifetime through substance use in financial, medical, relationship and occupational domains. For analysis purposes these were reduced to binary yes/no categories. Fifty-eight participants with ASD and 60 out of 76 control participants responded to alcohol problem questions. Regular smoking was reported as a binary yes/no variable, as was regular cannabinoid use, with complete data available for 79 participants with ASD and all controls. Regular cannabinoid use was reported as a binary yes/no variable and completed by 31 participants with ASD and 25 control participants. Usage of other street drugs was recorded as an initial binary yes/no variable and, if positive, through a multiple-choice question incorporating common UK street drugs. Eighty participants with ASD and all controls responded to the initial question, with 13 participants with ASD and 13 controls giving further answers.

### Phenotype statistical analysis

Statistical analysis was performed with IBM SPSS Statistics version 23 for Windows.^18^ Prevalence of comorbid psychiatric disorders was analysed through descriptive statistics for mean, s.d., variance and range. No comparative statistics of comorbid psychiatric diagnosis and associated medication usage was possible as by definition our control population was unaffected. Prevalence of sociodemographic, physical health comorbidity and family history was initially graphed. For normally distributed dependent variables, analysis was performed by *χ*²-test followed by a binomial logistic regression with ASD diagnosis (yes/no), with age and gender entered as covariates. Where dependent variables were non-normal, a non-parametric Mann–Whitney *U*-test was used, with Fisher's exact test for expected cell counts fewer than five.

### Genotyping

DNA was obtained from blood and saliva samples, and genotyping was carried out at the Medical Research Council Centre for Neuropsychiatric Genetics and Genomics (Hadyn Ellis Laboratory, Cardiff University, Wales). Samples were available from 90 individuals with ASD and 60 control participants.

Individuals were genotyped on two versions of the Illumina Infinium PsychArray: the Infinium PsychArray 24v1-2 (34 individuals with ASD, 59 controls) and the Infinium PsychArray with custom content (IPMCN PsychChip; 56 individuals with ASD, 1 control).

### CNV calling

CNVs were called using PennCNV run through a custom Galaxy pipeline.^19^^,^^20^ Individual samples were excluded if they had ≥30 CNVs, a waviness factor >0.03 or <−0.03 or a call rate <96%. Individual CNVs were excluded if their log R ratio (LRR) s.d. was >0.2. CNVs constituting <50 kb or >10 single nucleotide polymorphisms (SNPs) were removed, using a UNIX-based script before annotation (Supplementary Material); 373 samples remained after quality control. We annotated the CNVs called with a list of 53 CNVs associated with neurodevelopmental disorders (Supplementary Material).^21^^–^^23^ The breakpoints of the initial call of CNVs were inspected to confirm they met the CNV calling criteria. We required a CNV to cover more than half the critical interval and to include key genes in the region (if known), or in the case of single-gene CNVs, we required deletions to intersect at least one exon and duplications to cover the whole gene.^23^

### Polygenic risk scores

Genotype quality control was performed separately for polygenic risk scores (PRS), using the self-authored function genotypeqc in Stata. Full genotype quality control, genome-wide association study (GWAS) quality control and GWAS and genotype merging methods can be found in the Supplementary Material. After quality control, PRS were calculated on a subset of linkage independent markers (*r*^2^ < 0.2) generated using the –clump flag in PLINK. All risk scores were calculated based on the ‘risk’ allele, with weights for each risk allele taken from the GWAS beta-coefficient (calculated as the natural log of the GWAS odds ratio). PRS were calculated for ASD,^24^ attention-deficit hyperactivity disorder (ADHD),^25^ major depressive disorder^26^ and schizophrenia,^27^ using the –score flag in PLINK. Missing genotypes were scored using the mean imputation routine. PRS were calculated for the linkage independent markers with associations at ten *P*-value thresholds (*P* < 0.5, <0.1, <0.05, <10^−2^, <10^−3^, <10^−4^, <10^−5^, <10^−6^, <10^−7^ and <10^−8^). SNPs included in each model are available on request.

## Results

By definition, control participants did not have psychiatric morbidity and were not using any psychotropic medication.

### Psychiatric comorbidity

Comorbid psychiatric diagnosis was reported by 89.5% (*n* = 94) of individuals with ASD (Table 1). The most common comorbid diagnoses were depression (62.9%, *n* = 66) and anxiety (55.2%, *n* = 58); 44.8% of individuals with ASD reported dual depression and anxiety diagnoses. The mean BDI score for the participants with ASD was 20.356, which is on the border of mild (14–19) and moderate (20–28) depression severity. The mean for the control participants was 5.226, significantly different from the participants with ASD (*U* = 328.5, *Z*-score of −7.205, *r*=−0.68, *P* *<* 0.001), and within the minimal range (0–13). The significant association was seen across all BDI subscale scores except for question 19 (weight change).

**Table 1:** Psychiatric comorbidity as defined by ICD-10

ICD-10 code		Participants with ASD (*n* = 105)
	All comorbid psychiatric diagnosis	94 (89.5%)
**F30–39** Mood (affective) disorder	Any	69 (65.7%)
	Depression	66 (62.9%)
	Bipolar disorder	9 (8.6%)
	Hypomania	<3 (<2.9%)
**F40–48** Neurotic, stress-related and somatoform	Any	63 (60.0%)
	Anxiety	58 (55.2%)
	OCD	18 (17.1%)
	Phobias	9 (8.6%)
	Agoraphobia	8 (7.6%)
	Panic disorder	8 (7.6%)
	PTSD	6 (5.7%)
**F80–89** Psychological development	Any	30 (28.6%)
	Dyslexia	23 (21.9%)
	Dyspraxia	19 (18.1%)
**F90–98** Disorders with onset occurring in childhood or adolescence	Any	22 (21.0%)
	ADHD	20 (19.0%)
	Oppositional defiant disorder	<3 (<2.9%)
	Tic disorders	<3 (<2.9%)
**F20–29** Schizophrenia, schizotypal and delusional disorders	Any	13 (12.4%)
	Psychosis	12 (11.4%)
	Schizophrenia	4 (3.8%)
	Schizoaffective disorder	3 (2.9%)
**F10–19** Disorders due to substance use	Any	13 (12.4%)
	Alcohol misuse	10 (9.5%)
	Other substance misuse	9 (8.6%)
**F50–59** Syndromes associated with physiological disturbance	Any	8 (7.6%)
	Anorexia	4 (3.8%)
	Postnatal depression	3 (2.9%)
	Bulimia	<3 (<2.9%)
**F60–69** Disorders of adult personality	Any	4 (3.8%)
	Borderline personality Disorder	3 (2.9%)
	Other personality disorder	<3 (<2.9%)
**F00–09** Organic disorders	Pick's disease (frontotemporal dementia)	<3 (<2.9%)

Reported psychiatric comorbidity in participants with ASD grouped by ICD-10 classification. *n* indicates the number of individuals reporting the diagnosis; % indicates the percentage of the total amount of participants that responded.

ASD, autism spectrum disorder; OCD, obsessive–compulsive disorder; PTSD, post-traumatic stress disorder; ADHD, attention-deficit hyperactivity disorder.

### Psychotropic medication usage

The most widely prescribed medications among participants with ASD were antidepressants and anxiolytics, and over a quarter reported taking antipsychotics (Table 2).

**Table 2:** Psychotropic medication usage among participants with ASD

	Reported lifetime use, *n* (%)
Stimulants	6 (6.3%)
Antidepressants	74 (78.7%)
Anxiolytics	29 (31.2%)
Hypnotics	17 (18.1%)
Mood stabilisers	6 (6.5%)
Antipsychotics	25 (26.3%)

Reported psychotropic medication usage in participants with ASD based on the National Centre for Mental Health questionnaire response fields. *n* indicates the number of individuals reporting usage of medication of this type; % indicates the percentage of the total amount of participants that responded.

ASD, autism spectrum disorder.

### Sociodemographic phenotype

Data comparing sociodemographic phenotypes, physical health and family history for participants with ASD and controls are presented in Table 3. The average age of the participants with ASD was 37.8 years (s.d. 12.3) and for controls it was 40.7 years (s.d. 14.1, *P* = 0.89) because of matching. There were 80 males and 25 females among the participants with ASD (76.2% male), and 55 males and 21 females in the control participants (72.4% male, *P* = 0.56). Adults with ASD were significantly less likely to be currently working (odds ratio 0.174, *P* <0.001), to be married or cohabiting (odds ratio 0.29, *P* = 0.002), to be currently off work because of sickness or disablement (odds ratio 69.305, *P* <0.001) and to have alcohol-related problem (odds ratio 6.24, *P* = 0.001). No differences were found in rates of having one or more biological children.

**Table 3:** Physical health comorbidity, family history, social demographics and substance use in participants with ASD and matched controls

	Participants with ASD (*n*, %)	Matched controls (*n*, %)	Odds ratio (95% CI)	*P* value
Currently working	31, 32.0%	53, 72.6%	0.174 (0.09–0.35)	<0.001
Currently not working because of sickness/disablement	48, 49.5%	1, 0.01%	69.305 (9.23–520.42)	<0.001
Biological children	36, 35.6%	41, 54.0%	0.526 (0.26–1.05)	0.068
Married or cohabiting	49, 50.5%	58, 76.3%	0.29 (0.14–0.64)	0.002
Regular smoker	34, 43.0%	35, 46.1%	1.017 (0.99–1.04)	0.169
Problems due to alcohol use	21, 36.2%	5, 8.3%	6.42 (2.20–18.72)	0.001
Regular cannabinoids use	22, 27.9%	16, 21.1%	1.00 (0.97–1.03)	0.903
Problems due to cannabinoid use	9, 29.0%	4, 16.0%	1.02 (0.96–1.08)	0.590
Use of other street drugs	10, 12.5%	4, 5.3%	0.99 (0.95–1.04)	0.711
Problems due to other street drugs use	4, 33.3%	1, 7.7%	0.98 (0.89–1.08)	0.682
Physical health comorbidity				
Respiratory	36, 34.3%	16, 21.1%	1.84 (0.91–3.71)	0.089
Neurological	42, 40.0%	14, 14.0%	2.83 (1.40–5.74)	0.004
Metabolic	18, 17.1%	8, 18.4%	1.66 (0.67–4.11)	0.269
Rheumatological and orthopaedic	12, 11.4%	3, 3.9%	3.06 (0.82–11.33)	0.095
Cardiological	17, 16.2%	6, 7.9%	2.09 (0.77–5.66)	0.148
Renal and gastrointestinal	5, 4.8%	4, 5.3%	0.91 (0.23–3.59)	0.896
Oncological	1, 1.0%	0, 0%	–	0.996
Family history				
FHx ADHD	14 (14.1%)	9 (12.2%)	1.22 (0.49–3.02)	0.674
FHx bipolar disorder	9 (9.3%)	11 (15.1%)	0.58 (0.22–1.51)	0.265
FHx schizophrenia	6 (6.3%)	3 (4.1%)	1.58 (0.38–6.60)	0.529
FHx dementia	22 (22.4%)	16 (22.5%)	1.01 (0.48–2.11)	0.977
FHx PTSD	5 (5.1%)	2 (2.7%)	1.80 (0.34–9.67)	0.491
FHx autism	30 (34.1%)	16 (21.6%)	2.16 (1.03–4.53)	0.041

Reported physical health comorbidity, family health history, social demographics and substance use in participants with ASD based on the National Centre for Mental Health questionnaire response fields. The comparison group are matched healthy controls. For normally distributed samples, analyses were binomial logistic regression, with age and gender as covariates. Where data were non-normally distributed, the non-parametric Mann–Whitney *U*-test was used, with Fisher's exact test for expected cell counts fewer than five. *n* indicates the number of individuals; % indicates the percentage of the total amount of participants that responded.

ASD, autism spectrum disorder; FHx, reported family history; ADHD, attention-deficit hyperactivity disorder; PTSD, post-traumatic stress disorder.

### Physical health comorbidity

Adults with ASD were more likely to have neurological problems than controls (odds ratio 2.83, *P* = 0.004). *Post hoc* analysis within the neurological subgroup explored whether differences in the rates between participants with ASD and controls could be explained by comorbid epilepsy and seizure disorder, conditions reported to co-occur at elevated rates paediatric ASD populations. This demonstrated that the effect was predominantly because of an increased reported rate of migraine in individuals with ASD. Forty-one (42.7%) individuals with ASD reported lifetime history of migraine headaches compared with 15 (20.5%) control participants (odds ratio 2.60, *P* = 0.012, 95% CI 1.24–5.44). Eight individuals with ASD reported a lifetime history of epilepsy and seizure disorder compared with three control participants (odds ratio 2.15, *P* = 0.273, 95% CI 0.55–8.49). A further association between migraine headaches and epilepsy and seizure disorders was confirmed (odds ratio 11.38, *P* = 0.028).

### Family history of psychiatric disorder

Reported family history of autism was seen at a greater rate among participants with ASD (OR = 2.16, *P* = 0.041), but no differences were found for family history of ADHD, bipolar disorder, schizophrenia, dementia or post-traumatic stress disorder.

### Genetic analysis

#### CNV analysis

The 53 neurodevelopmental CNVs tested for were present in 3.8% (*n* = 4) of participants with ASD and 1.3% (*n* = 1) of controls. The CNVs in participants with ASD were 2q13 deletion (*n* = 2), 15q13.3 duplication (*n* = 1) and 16p13.11 duplication (*n* = 1). A single 2p16.3 deletion was found in one control.

#### Analyses of PRS

**Fig. 1:**
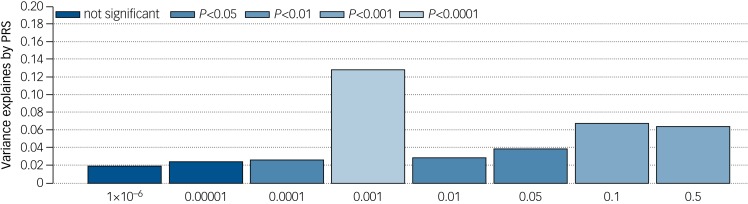
Percentage variance explained by PRS at analysed association levels for ASD. Percentage variance at eight association marker levels derived from linkage independent markers in the ASD genome-wide association study. Significance of associations between single nucleotide polymorphisms and ASD range from 0.5 to 1 × 10^−6^. Probability of association to be found at each individual variance level is denoted by *P* value.

ASD, autism spectrum disorder; PRS, polygenic risk score.

PRS derived from GWAS of ASD, ADHD, major depressive disorder and schizophrenia were calculated for each individual. Only the PRS derived from the ASD GWAS showed differences between cases and controls (Fig. 1); despite the modest sample size of this cohort, we calculate that approximately 12.86% (*P* < 0.00001) of variance can be explained from PRS derived from linkage independent markers showing association at *P* < 0.001 in the ASD GWAS (433 SNPs in model). No significant difference between cases and controls was seen for PRS for ADHD, major depressive disorder or schizophrenia.

## Discussion

In this study we report on the phenotypic characteristics and genetic profiles of a sample of individuals with ASD diagnosed in adulthood without intellectual disability. We found high rates of psychiatric comorbidity, problem alcohol use and medication usage in individuals with ASD. These individuals also had higher rates of neurological comorbidity than controls and there was an association between ASD diagnosis and migraine. There was a significant association between PRS for ASD and a diagnosis of ASD, but no significant increase in rate of rare neurodevelopmental CNVs in individuals with ASD.

### Comorbidity

A total of 89.5% of participants with ASD reported a further lifetime psychiatric comorbidity, comparable with previously reported populations including those diagnosed as adults or with intellectual disability (69–80%).^7^^–^^10^ Depression was the most common lifetime comorbid diagnosis with a rate of 62.9%, comparable with previously reported rates (53–70%) in larger samples including those diagnosed as children or with intellectual disability.^7^^,^^12^^,^^28^^,^^29^ Lifetime anxiety diagnosis rates of around 50% were also similar to previously reported in those with ASD but with intellectual disability.^3^^,^^8^^–^^10^^,^^30^ The implication that a primary diagnosis of ASD is linked to high lifetime rates of anxiety and depression even in populations without intellectual disability is important for clinical consultations. Whether the aetiology of ASD predisposes for depression and anxiety, or life events and difficulties experienced by individuals because of their ASD precipitate depression and anxiety, or both, is a ‘chicken and egg’ conundrum worthy of further study.

Rates of dyslexia (21.9%), dyspraxia (18.1%) and ADHD (19.0%), although elevated compared to the general population, were lower than in previous studies of ASD, which have mainly been in children.^5^^,^^7^^,^^31^ As our population were diagnosed as adults, fewer of the neurodevelopmental features that prompt assessment may be expected, and such a profile fits with genetic findings. Shared aetiology may explain the significantly higher rates of neurological conditions seen in participants with ASD, predominantly due to a strong prevalence of comorbid migraine. Although a pathophysiological link has been suggested for this in the past, this is the first clear evidence of an association, to our knowledge, and warrants further investigation.^32^^,^^33^

Lifetime psychotropic medication usage was concordant with the diagnosis findings. Nearly 80% of the population had taken antidepressant medications during their lifetime, greater than the rates predicted in the literature.^9^^,^^30^ Further, 26.3% reported taking antipsychotic medication when only 12.4% had a diagnosis of psychosis. This may be partly due to antipsychotic medication usage among four of the nine individuals reporting bipolar disorder, or for off-label use for symptomatic or behavioural management as is evidenced in adolescents and young adults.^9^^,^^34^

### Social demographics

The approximate 3:1 male:female ratio seen in our population was consistent with reported gender differences.^2^^,^^5^^,^^31^^,^^35^ Although alcohol and other substance use and smoking rates in our control and participants with ASD were broadly similar, ASD was associated with higher rates of problematic alcohol use. The reasons were unclear, but could be usage to self-medicate for the aforementioned anxiety as suggested by other authors, or to facilitate social interactions.^5^

Adults with ASD were found to be more than five times less likely to be employed individuals, with most reporting they were unemployed or unable to work because of illness. This suggests supportive employment is available for too few.^5^ We hypothesise that difficulties with social communication underlie the strikingly lower rate of marriage for those with ASD; however, interestingly, the difference in rates of biological children was not significant. This points to the clear impact of ASD even when diagnosed in adulthood, and the contribution of comorbid psychiatric conditions to the life experience of an individual with ASD.

### Genetic risk

Previous studies have demonstrated the strong heritability of ASD.^5^ As expected, we demonstrated that adults with ASD were significantly more likely to have family members with ASD. There were no other significant associations with mental health disorder family history. This result may be of use for patients presenting to clinical practice wanting to know associated family risks, but requires confirmation from a larger study. Participants with ASD had a slightly higher number of neurodevelopmental disorder–associated CNVs than controls, although this was not statistically significant in this sample. Although a statistically significant increase in burden of CNVs in this group might be confirmed in a larger population, rates were markedly lower than those reported in paediatric ASD populations, who may have a greater overall neurodevelopmental symptom profile.^27^ PRS analysis demonstrated a significant contribution of polygenic load of ASD-associated common variants to risk in adult participants with ASD.^16^^,^^24^^,^^36^ It is noticeable that a significantly increased polygenic risk burden was detected even in this relatively small sample size. Taken together, these results suggest that adults presenting with ASD may have a lower burden of rare penetrant variants and a higher polygenic contribution of common risk alleles than childhood ASD populations, potentially reflecting less severe neurodevelopmental disability.

### Limitations

The sample used in this study was drawn from the NCMH database, a resource rich in phenotypic data, and participants consented for anonymised genetic analysis. NCMH recruits through multiple methodologies. Sixty-nine of our 105 individuals with ASD were recruited from secondary ASD diagnostic services, a help-seeking population, and therefore our results may overestimate comorbidity rates reflective of recruitment bias. The benefit of the extensive range of data is offset by clinical diagnoses being initially self-reported, necessitating a review of the individuals’ notes to confirm specific diagnoses by specialist clinicians. All ASD diagnoses in this study were confirmed against ICD-10 diagnostic criteria. This prevents analysis by symptom severity and lacks the clarity of diagnostic scoring systems or rating scales such as the Autism Diagnostic Interview – Revised (ADI-R) however, this reflects the pattern seen within mental health services, where diagnoses are often clinical and diagnostic tool usage varies. By design our control population did not have psychiatric comorbidities, and this lack of control comorbidity data prevented comparative statistical analysis. It is also likely that demographic data in this control cohort are skewed by this lack of psychiatric comorbidity away from the general population mean, which may inflate effect sizes. All comorbid diagnoses were required to have been made by a doctor, but were self-reported and therefore not standardised. As a descriptive study we benefited from the large quantity and extensive domains available from the NCMH data-set. Where possible, results were elicited through subgroup analysis to reduce test volume. Because of the number of variables tested, only the association with ‘not currently working’ and ‘off work because of sickness or disablement’ would remain significant following a conservative Bonferroni correction. Associations here reported therefore require further testing with a larger sample.

### Implications for services

In this study we provide comorbidity rates and social demographic information for adults presenting with ASD, with clinical utility for consultations in adult ASD diagnostic services. Our findings suggest that a majority of adults with ASD have psychiatric comorbidity and should be appropriately screened and managed. Additionally, clinicians should be aware of associated social demographic features, including the high rates of alcohol problem use and being out of work. Signposting toward, and integration with, third-sector organisations and services supporting adults with ASD is vital, and our results may inform these services toward the possible difficulties some diagnosed with ASD in adulthood may face. The significantly increased polygenic risk burden seen in our sample is a difficult concept to convey in a clinical environment. It is likely that for some individuals, genetic testing would provide an element of assurance and diagnostic clarity, and our results may assist in genetic counselling. For many the benefit of an ambiguous answer is questionable. This is an area where future work with larger population sizes promises better results. Our findings suggest that the polygenic risk burden is present in clinical samples, and ongoing advances may allow us to explain this fully, along with the associations and implications.

## Supplementary material

For supplementary material accompanying this paper visit https://doi.org/10.1192/bjp.2019.30. click here to view supplementary material
