# Supplementary material for: Autism spectrum disorder diagnosis in adults: phenotype and genotype findings from a clinically derived cohort
Source: Br J Psychiatry. 2019 Nov;215(5):647–53. doi: 10.1192/bjp.2019.30 (PMC6949119; doi:10.1192/bjp.2019.30)
Supplement: Supplementary file 1 [file S0007125019000308sup001.docx]

**Supplementary Material**

**Contents**

Diagnosis Source (Figure 1-3)

Demographic Information

Comorbid Measures – Physical Health

Comorbid Measures – Mental Health

Population Psychiatric Comorbid Rates (Table S1)

CNV Calling

CNV Quality Control (QC) Filtering

CNV Annotation (Table S2)

Polygenic Risk Score Methodology

GWAS Included (Table S3)

References

**Diagnosis Source**

Casenotes for all 172 individuals obtained from the NCMH Database in June 2016 were reviewed for evidence of diagnosis by specialist services, meeting ICD-10 criteria for ASD. Sixty-nine of those recruited had diagnoses made by clinicians working at the Cardiff University Adult ASD Diagnostic Service. Twelve individuals had a diagnosis made by secondary mental health services, confirmed on mental health medical record review. For 24 individuals’ diagnosis was confirmed on review of correspondence from the diagnosing team held by their GP. Sixty-seven individuals were excluded for reasons documented in Figure 1.


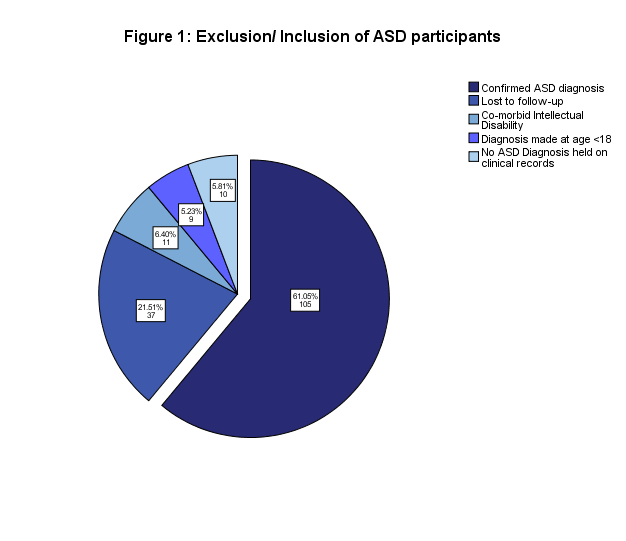


Our population was wholly self-reported Caucasian in origin, supported by HLA ancestry analysis demonstrating western European origin. One first degree family relative (parent-child) was present amount the ASD participants, along with a second-degree relative pairing (split between case and control), confirmed on genotyping (Figures 2 and 3).

Figures 2 and 3: Haplotype Ancestry Plots


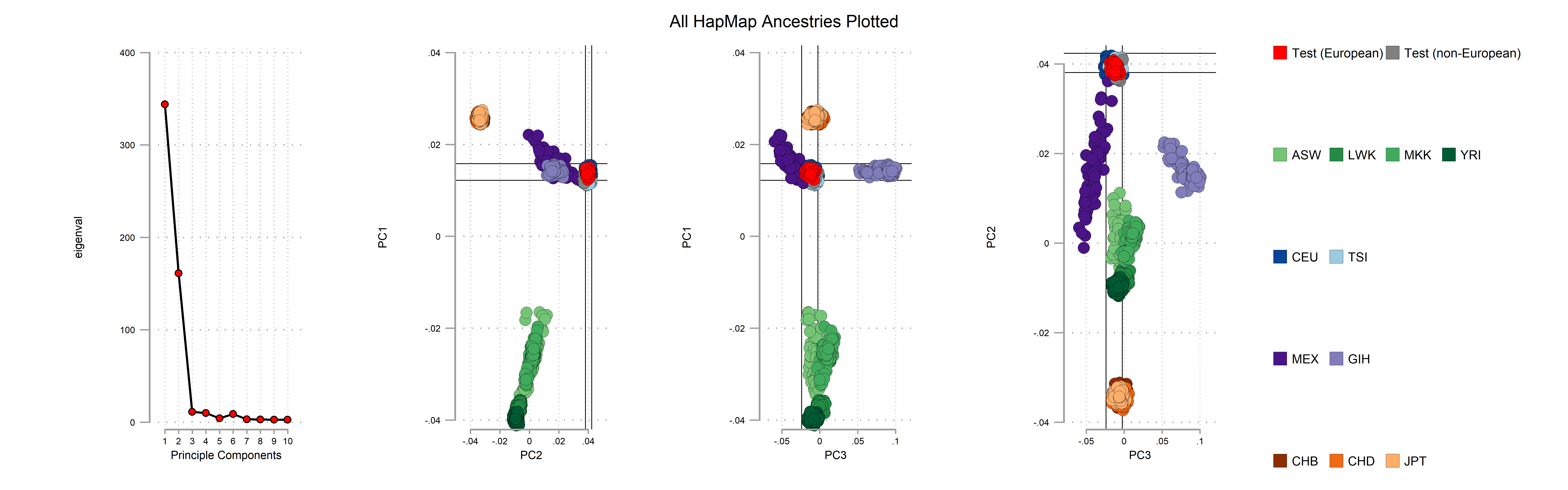


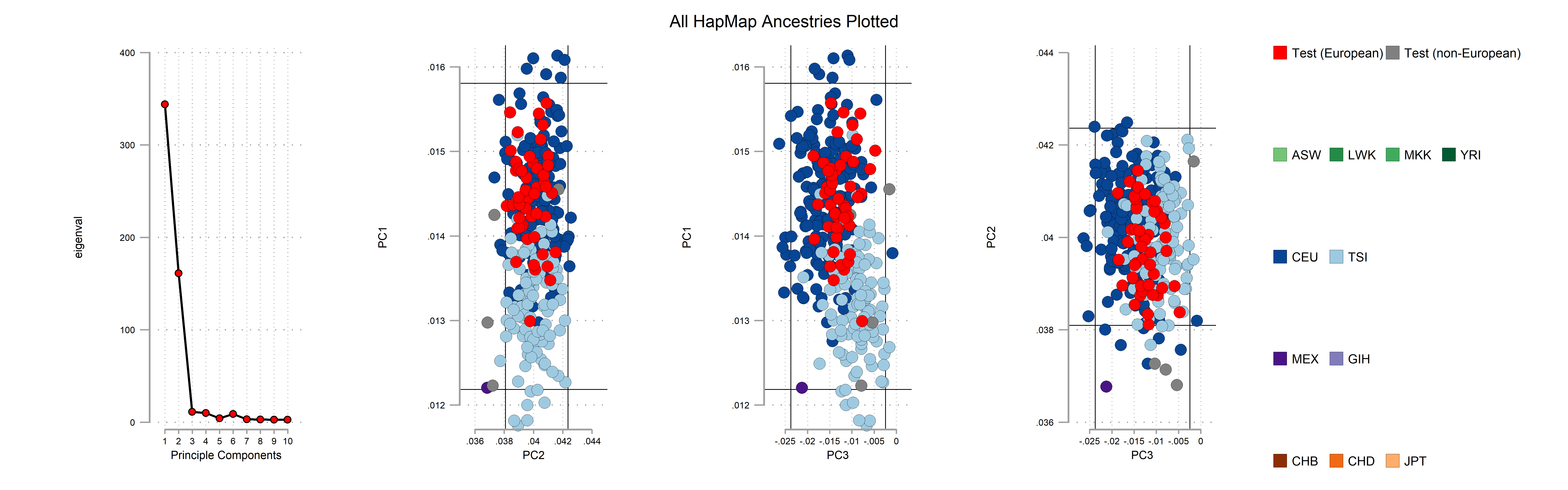


**Demographic Information**

Professions were coded into the following groups: 1= Legislator/ senior official/ manager, 2= Professional, 3= Technician/ associate professional/ civil servant, 4= Clerk/ office worker, 5= Service worker/ shop & market worker, 6= Skilled agricultural/ fishery worker, 7= Craft & related trade worker, 8= Plant & machinery operator/ assembler, 9= Elementary occupation, 10= Armed forces, 11= Not working due to sickness/ disablement, 12= Homemaker, 13= Full-time student, 14= Unemployed, 15= Retired, 16= Voluntary, 17= Other (free text). For analysis groups 1 to 10, 13, 16 and 17 were re-coded as currently working. Groups 11, 12, 14 and 15 were coded as not in work.

**Comorbid Measures – Physical Health**

The following 22 physical health comorbidities were reported: Asthma, Breast Cancer, Cancer (other), Diabetes Type 1, Diabetes Type 2, Elevated Lipids or Cholesterol, Epilepsy or Seizure Disorder, Gastric or Duodenal Ulcers, Heart Disease, Hypertension or High Blood Pressure, Kidney Disease, Liver Disease, Memory Loss or Dementia, Migraine Headache, Multiple Sclerosis, Osteoarthritis, Osteoporosis, Parkinson’s Disease, Rheumatoid Arthritis, Stroke or Haemorrhage, Overactive Thyroid or Hyperthyroidism, Underactive Thyroid or Hypothyroidism. These were grouped for analysis to: Oncological (Breast Cancer, Cancer (other)), Respiratory (Asthma), Neurological (Epilepsy or Seizure Disorder, Memory Loss or Dementia, Migraine Headache, Multiple Sclerosis, Parkinson’s Disease, Stroke or Haemorrhage), Metabolic (Diabetes Type 1, Diabetes Type 2, Elevated Lipids or Cholesterol, Overactive Thyroid or Hyperthyroidism, Underactive Thyroid or Hypothyroidism), Rheumatological or Orthopaedic (Osteoarthritis, Osteoporosis, Rheumatoid Arthritis), Cardiology (Heart Disease, Hypertension or High Blood Pressure), Renal or GI (Gastric or Duodenal Ulcers, Kidney Disease, Liver Disease). Data was absent in 5-6.6% of these fields, corresponding to 2-4 missing entries in controls and 7-9 missing entries in ASD participants.

**Comorbid Measures – Mental Health**

The 37 reported mental health diagnoses were: ADHD, Agoraphobia, Alcohol Abuse or Misuse, Alzheimer’s Disease, Anorexia, Anxiety, Asperger’s, Autism, Bipolar Disorder, Borderline Personality Disorder, Bulimia, Conduct Disorder, Dementia, Dementia with Lewy Bodies, Depression, Dyslexia, Dyspraxia, Early Onset Dementia, Frontotemporal Dementia or Picks Disease, Intellectual or Learning Disability, Mania or Hypomania, Obsessive Compulsive Disorder, Oppositional Defiant Disorder, Other Personality Disorder, Other Substance Abuse or Misuse, Panic Disorder, Parkinson’s Disease, Phobia, Postnatal Depression, Postnatal Psychosis or Puerperal Psychosis, Psychosis, PTSD, Schizoaffective Disorder, Schizophrenia, Tic Disorders, Tourette’s Disorder, and Vascular Dementia. All participants had responded to this question. The following ten comorbid psychiatric diagnoses were removed prior to analysis due to no recorded responses: Alzheimer’s Disease, Conduct Disorder, Dementia, Dementia with Lewy Bodies, Early Onset Dementia, Intellectual or Learning Disability, Parkinson’s Disease, Postnatal or Puerperal Psychosis, Tourette’s Disorder, and Vascular Dementia. Of note Intellectual Disability was screened out at the recruitment stage so therefore had no recorded responses by design. Population rates for these comorbidities drawn from the Adult Psychiatric Morbidity Survey 2014 are given in Table S1 (1).

**Table S1 – Population Psychiatric Morbidity Rates** (1)

| **Comorbidity** | **Population Rate** |
| --- | --- |
| ADHD | 9.7% |
| Agoraphobia | Not available |
| Alcohol Abuse or Misuse | 3.1% (AUDIT scores 16+) |
| Anorexia | Not available |
| Anxiety | 5.9% |
| Asperger’s | Not available |
| Autism | 0.8% |
| Bipolar Disorder | 2.0% |
| Borderline Personality Disorder | 2.4% |
| Bulimia | Not available |
| Depression | 3.3% |
| Dyslexia | Not available |
| Dyspraxia | Not available |
| Frontotemporal Dementia or Pick’s Disease | Not available |
| Mania or Hypomania | Not available |
| Obsessive Compulsive Disorder | 1.3% |
| Oppositional Defiant Disorder | Not available |
| Other Personality Disorder | 13.7% (Inc ASPD & BPD) |
| Other Substance Abuse or Misuse | 3.1% |
| Panic Disorder | 0.6% |
| Phobias | 2.4% |
| Postnatal Depression | Not available |
| Psychosis | 0.7% |
| Post-Traumatic Stress Disorder | 4.4% |
| Schizoaffective Disorder | Not available |
| Schizophrenia | Not available |
| Tic Disorders | Not available |

**CNV Calling**

Anonymised genotype datasets were stored as raw (CEL) files on a secure Linux server, and analysed with UNIX-based commands. The genotype calls, confidences, and summary files were processed with PennCNV software, run through a Galaxy pipeline(2)( http://penncnv.openbioinformatics.org/en/latest/user-guide/input/). Datasets were processed separately through the Galaxy pipeline dependent upon chip version of the Illumina Psych Chip. Calls used the hhall.hmm and gcmodel files obtained from PennCNV, and PsychChip or IPCMN -list files developed previously at Cardiff University. This generated canonical genotype clusters, Log R Rations (LRR), B Allele Frequencies and subsequent CNV calls (2,3).

**CNV Quality Control (QC) Filtering**

Distributions of QC parameters were examined in line with previous work. Individual samples were excluded if they had 30 or more CNVs, had a waviness factor (WF) >0.03 or <-0.03 or a call rate <96%. Individual CNVs were excluded if their LRR Standard Deviation was >0.2. CNVs constituting less than 50kb or >10 SNPs were removed utilising a UNIX based script prior to annotation. 373 samples remained after QC.

**CNV Annotation**

A list of 53 CNVs with statistically associated pathogenicity for neurodevelopmental phenotypes from two widely accepted sources previously developed by the authors was utilised (3–5). The CNVs selected are shown in Table S1. A script was initially used to annotate CNVS in the two output files. Positions of annotated CNVs were then manually inspected to ensure they covered the correct interval. Those not meeting criteria were excluded. Rules for CNV analysis were taken from previous work by the authors as specified in Table S2. Other researchers may choose to use other criteria for their annotation of CNVs.

**Table S2 – CNVs and Breakpoints – adapted from Kendall *et al*** (3)

| **CNV** | **Criteria** |
| --- | --- |
| 1p36 del/dup | Size >50% of critical region, affecting *GABRD* |
| TAR del/dup | Size >50% of critical region |
| 1q21.1 del/dup | Size >50% of critical region |
| *NRXN1 del* | Exonic deletions |
| 2q11.2 del/dup | Size >50% of critical region, affecting both *LMAN2L* and *ARID5A* |
| 2q13 del/dup | Size >50% of critical region |
| 2q13 del/dup (*NPHP1*) | Size >50% of critical region, affecting *NPHP1* |
| 2q21.1 del/dup | Size >50% of critical region |
| 2q37 del/dup (*HDAC4*) | Size >50% of critical region, affecting *HDAC4* |
| 3q29 del/dup | Size >50% of critical region |
| Wolf-Hirschhorn del/dup | Size >50% of critical region |
| Sotos Syn/5q35 dup | Size >50% of critical region |
| 6q16 del/dup (*SIM1*) | Exonic deletions; whole gene duplications |
| Williams Beuren Syn del/dup | Size >50% of critical region |
| 7q11.23 distal del/distal dup | Size >50% of critical region |
| 8p23.1 del/dup | At least 1Mbp of critical region |
| 9q34 del/dup (*EHMT1*) | At least 1Mbp CNVs, including *EHMT1* |
| 10q11.21q11.23 del/dup | Size >50% of critical region |
| 10q23 del/dup | At least 1Mbp, including *NRG3* and *GRID1* |
| Potocki-Shaffer Syn del/11p11.2 dup (*EXT2*) | Size >50% of critical region, including *EXT2* |
| 13q12 del/dup (*CRYL1*) | Exonic deletions; whole gene duplications |
| 13q12.12 del/dup | Size >50% of critical region |
| 15q11.2 del/dup | Size >50% of critical region |
| PWS del/dup | Full critical region, ~4Mbp |
| 15q11q13 del/dup BP3-BP4 | Size >50% of critical region |
| 15q11q13 del/dup BP3-BP5 | Size >50% of critical region |
| 15q13.3 del/dup | Size >50% of critical region |
| 15q13.3 del/dup (*CHRNA7*) | Size >50% of critical region, affecting *CHRNA7* |
| 15q24 del/dup | At least 1Mbp between the A-E intervals |
| 15q25 del/dup | At least 1Mbp between the A-D intervals |
| Rubinstein-Taybi del/dup (*CREBBP*) | Exonic deletions; whole gene duplications |
| 16p13.11 del/dup | Size >50% of critical region |
| 16p12.1 del/dup | Size >50% of critical region |
| 16p12.2-p11.2 del/dup (7.1-8.7 Mb) | Size >50% of critical region |
| 16p11.2 distal del/distal dup | Size >50% of critical region |
| 16p11.2 del/dup | Size >50% of critical region |
| 17p13.3 del/dup (*YWHAE*) | Exonic deletions; whole gene duplications |
| 17p13.3 del/dup (*PAFAH1B1*) | Exonic deletions; whole gene duplications |
| 17p12 del (HNPP)/dup (CMT1A) | Size >50% of critical region, affecting *PMP22* |
| Smith-Magenis/Potocki-Lupski Syn | Size >50% of critical region |
| 17q11.2 del/dup (*NF1*) | Size >50% of critical region, affecting *NF1* |
| 17q12 del/dup | Size >50% of critical region |
| 17q21.31 del/dup | Size >50% of critical region |
| 17q23.1q23.2 del/dup | Size >50% of critical region |
| 22q11.2 del/dup | Size >50% of critical region |
| 22q11.2 distal del/dup | Size >50% of critical region |
| *SHANK3* del/dup | At least 1Mbp CNVs, including *SHANK3* |
| “Large” CNVs | Size > 20Mbp + >50 genes |

***Polygenic Risk Score Methodology***

### Genotype quality control

Genotypes quality control (QC) was performed separately for the Infinium PsychArray and IPMCN-PsychChip using the self-authored function genotypeqc in Stata. Briefly, genotypes are aligned to genome build hg19 and renamed according to a reference panel (Haplotype Reference Consortium version 1.1). As is the case with the PsychArray, multiple assays may be present that capture the same SNP. Where more than one assay was present for a single marker, only one was retained and carried forward for analysis. Markers were excluded if they show allele frequency deviation from a matched ancestry reference panel (+/- 10% from 1000 genomes CEU subset). Individuals were excluded if they show evidence of excessive relatedness in line with genotyping error. Additional QC was then applied, including restriction to markers with minor allele count of at least 5, removal of markers with genotype missingness greater than 2%, removal of markers with Hardy-Weinberg equilibrium exact test statistics of P < 10^-10^, removal of individuals with excess or deficiency of heterozygosity (+/- 4x standard deviation of the sample mean), and removal of individuals with total genotype missingness greater than 2%.

### GWAS quality control

Summary GWAS data was downloaded from the Psychiatric Genomics Consortia <https://www.med.unc.edu/pgc/results-and-downloads>. Additional QC was performed using the self-authored function summaryqc in Stata. Briefly, markers were aligned to genome build hg19 and renamed according to a reference panel (Haplotype Reference Consortium version 1.1). As with the raw genotyping procedure, where more than one assay was present for a single marker, one was retained and carried forward for analysis.

Self-authored Stata packages available at <https://github.com/ricanney/stata>.

### GWAS and genotype merging

Prior to generating PRS scores for each GWAS, the genotype samples were restricted to those markers present in both genotyping arrays and the training GWAS. Allele codes for each dataset were assigned to a common map and strand. Markers showing strand incompatibilities and ambiguities were excluded, for example C/A genotype in dataset 1 and G/A in GWAS are incompatible on either strand and were excluded as were ambiguous genotypes where strand could not be confirmed (i.e. C/G or A/T genotypes).

The “restricted” genotype datasets were merged using the --bmerge flag in PLINK and ancestry covariates were derived using the --pca flag in PLINK. PCA was performed on a subset of linkage independent (r^2^ < 0.2) ancestry informative markers.

### Polygenic risk scoring

Polygenic risk scores (PRS) were calculated on a subset of linkage independent markers (r^2^ < 0.2) generated using the --clump flag in PLINK. All risk scores were calculated based on the “risk” allele, with weights for each risk allele taken from the GWAS beta-coefficient (calculated as the natural log of the GWAS odds ratio). PRS were calculated for ASD, ADHD, MDD and Schizophrenia using the --score flag in PLINK. Missing genotypes were scored using the mean imputation routine. PRS were calculated for the linkage independent markers with associations at ten p-value thresholds (P< 0.5, < 0.1, < 0.05, < 10^-2^, < 10^-3^, < 10^-4^, < 10^-5^, < 10^-6^, < 10^-7^ and < 10^-8^).

SNPs included in each model are available on request.

### Calculation of variance explained

Regressions were performed for the dependent variable ASD diagnosis, predicted by allele count, sex and 10 ancestry derived principle components, with and without PRS. The variance explained by PRS was calculated as the differences in the two models. The reported P-values that the two model are equal were estimated using ftest function in Stata.

***GWAS Included – Table S3***

| Diagnosis | Sample Size | Publication | Data Access / Download |
| --- | --- | --- | --- |
| Attention Deficit Disorder | 20,183 ADHD cases  35,191 controls | Demontis, Walters, Martin et al., Nat Genet. 2018 Nov; PMID: 30478444 | (ADHD_June2017)  <https://www.med.unc.edu/pgc/results-and-downloads/> |
| Autism Spectrum Disorder | 18,381 ASD cases  27,969 controls | Grove, Ripke, Als et al., Nat Genet. Accepted Dec 2018;  BioRxiv PrePrint: <https://www.biorxiv.org/content/early/2017/11/27/224774> | (IPSYCH-PGC ASD Nov2017)  <https://www.med.unc.edu/pgc/results-and-downloads/> |
| Major Depressive Disorder | 135,458 MDD cases  344,901 controls | Wray, Ripke, Mattheisen et al., Nat Genet. 2018 May;50(5):668-681. | (MDD2018)  <https://www.med.unc.edu/pgc/results-and-downloads/> |
| Schizophrenia | 36,989 SCZ cases  113,075 control | Schizophrenia Working Group of the Psychiatric Genomics Consortium. Nature. 2014 Jul;511(7510):421-427. | (SCZ2)  <https://www.med.unc.edu/pgc/results-and-downloads/> |

References:

1. Adult Psychiatric Morbidity Survey: Survey of Mental Health and Wellbeing, England, 2014 - NHS Digital [Internet]. [cited 2018 Dec 7]. Available from: https://digital.nhs.uk/data-and-information/publications/statistical/adult-psychiatric-morbidity-survey/adult-psychiatric-morbidity-survey-survey-of-mental-health-and-wellbeing-england-2014

2. Afgan E, Baker D, van den Beek M, Blankenberg D, Bouvier D, Čech M, et al. The Galaxy platform for accessible, reproducible and collaborative biomedical analyses: 2016 update. Nucleic Acids Res [Internet]. Oxford University Press; 2016 Jul 8 [cited 2017 Nov 5];44(W1):W3–10. Available from: https://academic.oup.com/nar/article-lookup/doi/10.1093/nar/gkw343

3. Kendall KM, Rees E, Escott-Price V, Einon M, Thomas R, Hewitt J, et al. Cognitive Performance Among Carriers of Pathogenic Copy Number Variants: Analysis of 152,000 UK Biobank Subjects. Biol Psychiatry [Internet]. 2017 Jul [cited 2017 Aug 6];82(2):103–10. Available from: http://linkinghub.elsevier.com/retrieve/pii/S0006322316327111

4. Coe BP, Witherspoon K, Rosenfeld JA, van Bon BWM, Vulto-van Silfhout AT, Bosco P, et al. Refining analyses of copy number variation identifies specific genes associated with developmental delay. Nat Genet [Internet]. Nature Research; 2014 Sep 14 [cited 2017 Oct 22];46(10):1063–71. Available from: http://www.nature.com/doifinder/10.1038/ng.3092

5. Dittwald P, Gambin T, Szafranski P, Li J, Amato S, Divon MY, et al. NAHR-mediated copy-number variants in a clinical population: mechanistic insights into both genomic disorders and Mendelizing traits. Genome Res [Internet]. Cold Spring Harbor Laboratory Press; 2013 Sep 1 [cited 2017 Oct 22];23(9):1395–409. Available from: http://www.ncbi.nlm.nih.gov/pubmed/23657883
